# Radioimmunotherapy in Oncology: Overview of the Last Decade Clinical Trials

**DOI:** 10.3390/cancers13215570

**Published:** 2021-11-07

**Authors:** Aurélie Rondon, Jacques Rouanet, Françoise Degoul

**Affiliations:** 1 Advanced Drug Delivery and Biomaterials, Louvain Drug Research Institute, UCLouvain, BE-1200 Brussels, Belgium; 2 Imagerie Moléculaire et Stratégies Théranostiques, Inserm UMR1240, Université Clermont-Auvergne, F-63000 Clermont-Ferrand, France; jrouanet@chu-clermontferrand.fr; 3 Service de Dermatologie et d’Oncologie Cutanée, CHU Estaing, F-63011 Clermont-Ferrand, France; 4 CNRS 6293, INSERM U1103, GReD, Centre de Recherche et de Biologie Clinique, Université Clermont-Auvergne, F-63000 Clermont-Ferrand, France

**Keywords:** RIT, hematologic cancers, solid cancers, antibody fragments, PRIT, radionuclides

## Abstract

**Simple Summary:**

Monoclonal antibody-bearing radionuclides have been under clinical investigation over the last two decades for their use in theranostic (diagnostic and therapeutic) applications in cancer. However, despite the numerous trials that have been conducted, only two radioimmunotherapies (RIT) have been approved by the FDA for the targeted therapy of hematologic tumors expressing CD20 antigens. Moreover, RIT applications for solid cancers faced major issues—such as radiotoxicity due to low antibodies penetrance requiring substantial curative dose—where new discoveries concerning antibody engineering or radionuclides are trying to overcome. Here, we performed an overview of the last 11-year clinical trials involving RIT for solid and non-solid cancers conducted either with full antibodies or antibody fragments. We discussed the low-to-moderate efficiency of RIT compared to conventional therapies and described the last advances in clinic for antibodies carriers (F(ab′)_2_, Fab′, ScFv). Finally, we discussed about the complexity of RIT as a therapy and depicted both the issues and the prospects of such a strategy.

**Abstract:**

The specific irradiation of tumors with selective radiolabeled antibodies constitutes an attractive therapeutic approach. Consequent preclinical research has been conducted by both biologists to identify pertinent targets and to select corresponding antibodies (mAb) and by radiochemists to radiolabel mAbs. These numerous preclinical investigations have ascertained the therapeutic interest of radioimmunotherapy (RIT) protocols in mice models. Here, we summarize the clinical studies that have been performed the last decade, including clinical trials (phases I, II, and III), prospective and retrospective studies, and cases series. We thereby reported 92 clinical studies. Among them, 62 concern the treatment of hematological malignancies, and 30 concern solid tumors. For hematologic diseases, the analysis was complex due to the high discrepancy of therapeutic strategies (first-line therapy, consolidation, stem cell transplantation conditioning) as well as the high variety of malignancies that were treated. The clinical studies from the last decade failed to expand anti-CD20 RIT indications but confirmed that RIT using radiolabeled anti-CD20 remains a pertinent choice for patients with relapse follicular lymphomas. For solid tumors, the positive benefit of RIT is more mitigated, apart for few malignancies that can be treated locally. Clinical trials also demonstrated the potential of some antibody formats, such as F(ab′)_2_, which has already been approved by the China State FDA under the trend name Licartin®. Despite disparate results, mAb fragments are an interesting prospect for the improvement of RIT efficiency as well as for pretargeted strategies that delay the injection of radioactive treatments from the mAb ones.

## 1. Introduction

When addressing the role of radionuclides and their action toward tumor antigens, the use of monoclonal antibodies (mAbs) as vectors has been a great challenge since the 1980s. The idea, which was initially conceptualized in 1900 by Erlich (“magic bullet”) [1], combined the therapeutic properties of radioisotopes with specific vectors in order to eradicate tumors, regardless of their location. The proof of concept of radioimmunotherapy (RIT) was then demonstrated in preclinical models since the 1970s thanks to the development of hybridomas by Kohler and Milstein [2]. Several studies performed thereafter with radiolabeled antibodies confirmed the potential of RIT in mice, as RIT decreased tumor growth and/or improved survival [3,4].

One of the main prerequisites for RIT relies on the accessibility of the antigens by their cognate mAbs. Therefore, membrane proteins such as certain specific activating proliferation receptors (HER family members), the hematopoietic cluster of differentiation proteins (CD20 in most cases), or the carcinoma embryonic antigen (CEA) were considered to be antigens of interest for such an approach. However, it should be noted that these antigens are not exclusively found in tumors, so targeting them may induce adverse and unwanted events. Such off-targeted effects mainly depend on the radionuclide toxicity, and they can induce hematopoietic damage due to the long half-life of mAbs in the blood (~7- to 21-day half-life depending on the isotype).

An increasing panel of radionuclides with different properties (half-life, spectra emission, particles or electrons) is currently under evaluation in theranostic approaches (for a review see [5]). However, until now, the choice of radionuclides for RIT in clinical trials was limited to I-131 (8.0 days half-life), Y-90 (2.7 days half-life), Lu-177 (6.7 days half-life), and Re-188 (16.9 hours half-life) for β^-^-emitters and to Bi-213 (45.6 minutes half-life) and At-211 for α-particles (7.2 hours half-life). As therapeutic radionuclides aim to destroy the tumor, the dose that is delivered should induce enough lethal alterations to prevent DNA reparations and escape mechanisms. Dosimetry determination is crucial for RIT and can be calculated by combining the intrinsic radionuclide theoretical properties and radiolabeled mAb pharmacokinetics. In some cases, a personalized dosimetry can be performed using a couple of radionuclides (one for imaging, one for therapy) that are bound to the same binding-site on the mAb or by using therapeutic isotopes that are also able to emit γ-rays for SPECT acquisition, such as I-131 or Lu-177. Even though dosimetry is now well established for external beam radiotherapy, it is still challenge to accurately determine it in RIT [6]. RIT delivers continuous ionizations over time, which are characterized by the linear energy transfer (LET): LET (~0.2 keV/µm) for β-emitters and a high LET (~50–230 keV /µm) for α-particles [7].

**Figure 1:**
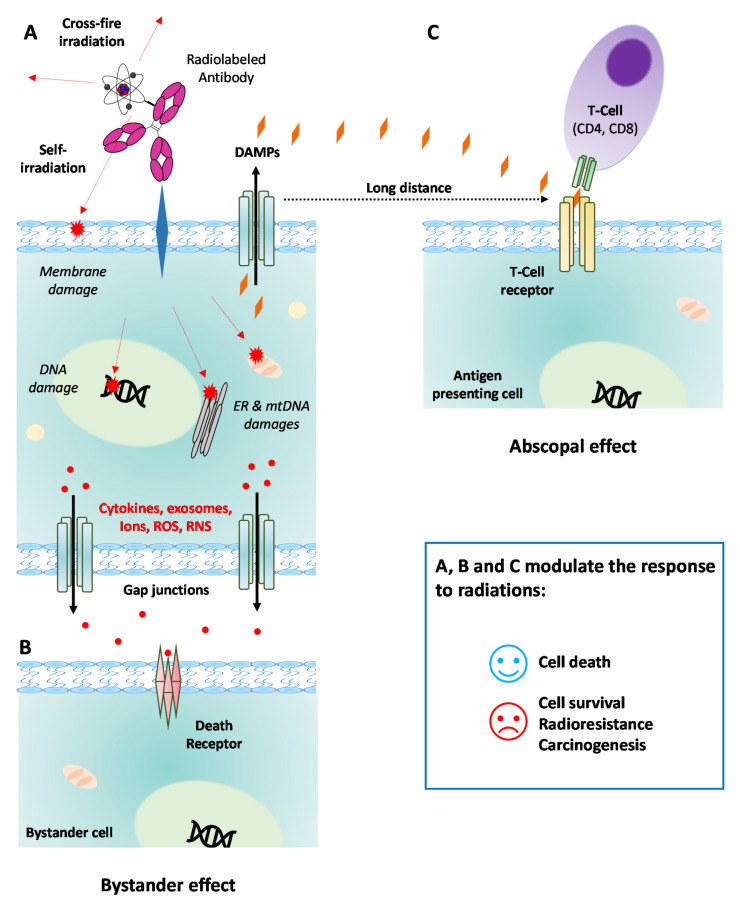
Cell mechanisms underlying radioimmunotherapy (RIT). (**A**) Targeted effects on tumor cell after binding by a radiolabeled mAb to its cognate membrane antigen. RIT induces direct damages to DNA, mtDNA, membranes, and other cell components (e.g., ER). Surrounding cells are also directly irradiated through the crossfire effect. Damages to the cell lead to the secretion of cytokines, ions, ROS, RNS, or exosomes that are released in the extracellular microenvironment. (**B**) Off-target bystander effect induced in other cancer cells (close or not). Cytokines and other death effectors released in the microenvironment bind to the cell death receptors. (**C**) Off-target abscopal effect involving the immune system response far away from the irradiated cells. DAMPs excreted by the irradiated cell can bind the T-cell receptor of an antigen-presenting cell, resulting in the activation of the immune system through the binding of the CD4 or CD8 T lymphocytes. NB: Similar mechanisms occur if RIT is performed with a radiolabeled mAb that can be internalized into the cell. DAMPs: damage-associated molecular patterns; ER: endoplasmic reticulum; mAb: monoclonal antibody; mtDNA: mitochondrial DNA; RNS: reactive nitrogen species; ROS: reactive oxygen species.

**Figure 2:**
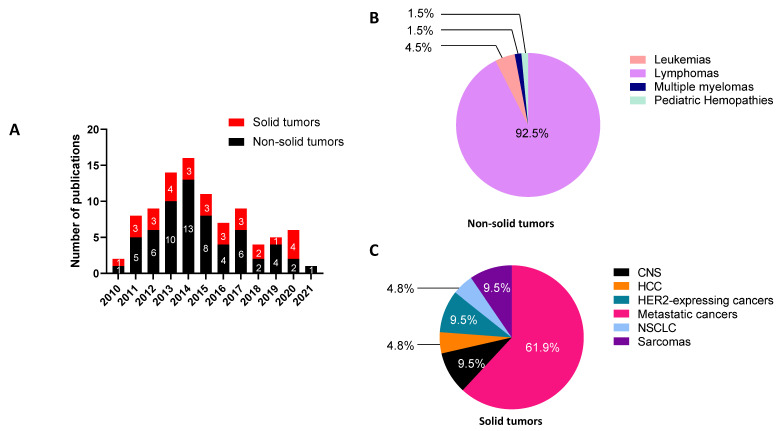
Publications about RIT clinical trials from 2010 to 2021. (**A**) Number of publications per year reporting clinical trials with RIT protocols for solid and non-solid tumors. (**B**) Repartition of clinical trials for non-solid cancer for the entire period of 2010–2021. Leukemias encompass acute myeloid leukemias and acute lymphoblastic leukemias; lymphomas encompass follicular lymphomas, mantle cell lymphomas, Burkitt lymphomas, diffuse large B-cell lymphomas, marginal zone lymphomas, and Hodgkin lymphomas. (**C**) Repartition of clinical trials for solid cancers for the entire 2010–2021 period. HER2-expressing cancers encompass breast cancer, peritoneal carcinomatosis, and gastric cancer; metastatic cancers were from colorectal cancers, prostate cancers, melanoma, pancreatic carcinomas, and renal cell carcinomas. CNS: central nervous system tumors (medulloblastomas and neuroblastomas); HCC: hepatocellular carcinoma; NSCLC: non-small cell-lung cancer.

Besides the intrinsic properties of radionuclides, RIT also induces different therapeutic effects depending on the properties of the antibody that is used (Figure 1). A radiolabeled antibody that undergoes internalization leads to more DNA double-strand breaks than a non-internalizing antibody targeting the cell membrane does. A previous review discussed the effects of (1) crossfire irradiation, which induces the irradiation of juxtracrine cells (Figure 1A); (2) bystander effects, which occur through communication between targeted cells and the surrounding cells (Figure 1B); and (3) the abscopal effect, which is characterized by the death of non-targeted cells that are distant from the targeted cells (Figure 1C) [6,8]. These three processes result in oxidative stress followed by cell death following macromolecule alterations. This involves the activation of numerous pathways as well as the immune response due to neoantigen occurrence for abscopal response [8]. RIT thus represents an attractive therapeutic approach for disseminated tumors. In this work, we wanted to provide an overview on the clinical evaluations of RIT that have taken place over the last decade. We focused on the clinical studies that have been reported since the first review by Pouget et al. published in 2011 [7].

The web search concerning publication from the last 10 years was performed on PubMed using the terms “Radioimmunotherapy” and both the filters “Clinical Trials” and “10 years”. Over the 129 publications that were retrieved, we removed 35 that were irrelevant (i.e., imaging studies only, dosimetry calculations only, or external radiotherapy combined with immunotherapy). For the ongoing clinical trials, we searched the terms “Radioimmunotherapy” and “radiolabeled antibody” on government websites from the US, Europe, China, and Japan as well as the WHO ICTRP. Here, we report and discuss RIT efficacy for both hematological and solid tumors based on clinical trial data as well as prospects based on the use of fragments or pretargeting strategies.

## 2. Last Ten Years Publications Involving RIT Protocols

We identified 92 publications describing RIT in humans that have been published in the last 10 years (Figure 2). Among them, we observed that 67% concerned RIT for non-solid tumors (62 studies) and that only 33% concerned the use of RIT for solid tumors (30 studies), as detailed in Figure 2A. The years 2013 and 2014 were the most prolific, with 30 out of the 92 publications being published during the last decade. The repartition of non-solid cancer RIT (Figure 2B) showed that studies on lymphomas (encompassing follicular lymphomas, mantle-cell lymphomas, Burkitt lymphomas, diffuse large B-cell lymphomas, marginal zone lymphomas, and Hodgkin lymphomas) were highly preponderant, thus representing 92.5% of the investigations. In contrast, the RIT of solid tumors has been assessed on a higher variety of targets and tumors (Figure 2C), with metastatic cancers representing 61.9% of the reported studies. In the following parts, we will discuss the selection of the RIT strategy depending on whether it is for the treatment of solid or non-solid cancers, antigen targets, association protocols, and the choice of mAbs or radionuclides.

### 2.1. Combination for Hematologic Malignancies: Modest Outcomes-Based RIT?

During the first decade of the 2000s, positive clinical results led to the subsequent FDA-approval of two radioimmunoconjugates, ^90^Y-ibritumomab tiuxetan (Zevalin®, Bayer, Leverkusen, Germany) in 2002 and ^131^I-tositumomab (Bexxar ®, GSK, Brentford, UK) in 2003, both of which are based on murine anti-CD20. This progress then opened the door widely for RIT for hematologic malignancies [9]. Both were initially indicated for the treatment of patients with relapsed or refractory (R/R), low-grade or follicular B-cell non-Hodgkin lymphoma (B-NHL), including patients with rituximab refractory follicular NHL. In 2009, ^90^Y-ibritumomab tiuxetan obtained an indication for the consolidation strategy for patients with previously untreated follicular NHL who had achieved a partial or complete response to first-line chemotherapy.

After the first approval, a significant number of clinical trials (62 studies over the 2010–2021 period, summarized in Table 1 for anti-CD-20 RIT and in Table 2 for other approaches, for details see Supplementary Table S1) evaluated the interest of RIT in various hematologic malignancies and therapeutic strategies. Most studies (~90%) assessed the efficacy of anti-CD-20 radioimmunoconjugates (^90^Y-ibritumomab tiuxetan: *n* = 36, ^131^I-tositumomab: *n* = 12, radiolabeled rituximab: *n* = 6). Among them, the majority concerned B-NHL patients, including one-third with a follicular lymphoma (FL). Diffuse large B-cell lymphomas (DLBCL), mantle-cell lymphoma (MCL), marginal-zone lymphoma (MZL), and Burkitt lymphoma were less represented. Only five studies reported other hematologic diseases such as acute lymphoblastic leukemia (ALL, *n* = 1), acute myeloid leukemia (AML, *n* = 2), Hodgkin lymphoma (*n* = 1), pediatric hematologic malignancies and non-malignancies (*n* = 1), and multiple myeloma (MM, *n* = 1). Interestingly, phase II trials represented 50% of the studies, phase I and I/II trials represented 22% of the studies, and phase III trials only represented 10%. The rest of the reported studies were prospective, retrospective, or cases series.

#### 2.1.1. Therapeutic Strategies Studied

Concerning B-NHL, the clinical trials evaluated RIT efficacy for relapsed/refractory (R/R) malignancies (28%) and in consolidation (32%) or conditioning (27%) strategies, and only 12% presented evaluations of RIT as a first-line therapy. One study evaluated RIT as a late intensification strategy. RIT was mostly used in combination with the corresponding cold mAb (85%) in order to saturate non-neoplastic CD-20 receptor sites and to limit toxicities. When used as a consolidation treatment, either in first-line or in R/R patients, RIT was associated with cold rituximab (R-) and with chemotherapy 89% of the time. Chemotherapy protocols were fludarabine/mitoxantrone/rituximab (FMR) for FL or MZL [10,11,12,13]; cyclophosphamide/doxorubicine/vincristine/predisolone (CHOP) [24,25,31,53,54,57,66]; cyclophosphamide/vincristine/predisone (CVP) [26]; prednisolone/etoposide/chlorambucil/lomustine (PECC) [32]; or etoposide/prednisolone/vincristine/cyclophosphamide/doxorubicine (EPOCH) [58] for more aggressive lymphomas. These chemotherapy protocols corresponded to protocols that are commonly used in clinical practice, and in most cases, RIT was an adjuvant therapeutic in these protocols. It must be noted that one case series showed that RIT using ^90^Y-ibritumomab tiuxetan could be beneficial in a consolidation protocol for patients with aggressive NHL who did not fully respond to autologous stem cell transplantation (Auto-SCT) (RIA 2011). In the last decade, RIT has also been evaluated as a part of the conditioning protocol before auto-SCT (*n* = 13) or allo-SCT (*n* = 5) in patients presenting untreated [69,70] or R/R [14,27,28,33,36,37,38,39,40,45,47,59,60] or transformed [34] B-NHL. Conditioning protocols use high-dose carmustine/etoposide/cytarabine/melphalan (BEAM) [27,28,33,34,38,59] or reduced-intensity conditioning (fludarabine alone [60], fludarabine/melphalan/alemtuzumab [36], or fludarabine/melphalan/thiothepa [37]) without or with total-body-irradiation [39,40]. In 75% of cases, the conditioning protocols were associated with anti-CD20 cold mAb. As a first-line strategy, anti-CD20 RIT was used without combination with CT but was systemically associated with a cold anti-CD20 mAb [12,15,16,17,22,46].

#### 2.1.2. Low-Grade B-NHL

The efficacy and good tolerance of ^90^Y-ibritumomab tiuxetan [12,15,16] and ^131^I-rituximab [46] have been demonstrated in first-line therapy for advanced FL. However, to the best of our knowledge, this was not followed by further phase III evaluation. Phase II evaluations with ^90^Y-ibritumomab tiuxetan for first-line consolidation protocols in FL [10,11] showed promising results and were followed by a phase III study [18]. A clear advantage in progression-free survival (PFS) compared with no consolidation strategy was thereby demonstrated. However, phase III first-line consolidation studies using ^131^I-tositumomab failed to demonstrate a PFS advantage compared to cold rituximab consolidation strategies [53,54]. In the relapse/refractory consolidation strategy, RIT was also able to provide a clinical benefit, as suggested for ^90^Y-ibritumomab tiuxetan in a phase II trial [19,20].

Concerning R/R FL, the safety and efficacy of ^90^Y-ibritumomab tiuxetan has been demonstrated in a phase I study [21]. Phase III trials otherwise assessed RIT with ^131^I-tositumomab. However, this trial has been early terminated due to a lack of feasibility, as only 14 patients have been included, thereby failing to draw significant conclusions [55].

In MZL patients, three Phase II studies suggest that ^90^Y-ibritumomab tiuxetan could also have benefits in first-line [17,22] or consolidation therapy [13].

#### 2.1.3. Aggressive B-NHL

In aggressive B-NHL encompassing transformed low-grade B-NHL, DLBCL, and MCL, anti-CD20 RIT was evaluated either for conditioning protocols before SCT or for response consolidation in untreated or R/R patients. Phase II and retrospective studies performed during a conditioning regimen followed by Auto-SCT or Allo-SCT showed that anti-CD20 RIT was well-tolerated [27,33,34,36,37,39]. A significant benefit in survival was highlighted in most of the studies evaluating anti-CD20 RIT regardless to the type of aggressive B-NHL [33,34,38,39,40,47,60]. However, in MCL, one study failed to show a significant improvement in survival compared to protocols involving cold rituximab [27]. Despite those promising results, the Phase III trial comparing conditioning ^131^I-tositumomab-associated BEAM or cold rituximab did not show any differences neither in terms of PFS nor in the overall survival (OS) rates for patients with chemotherapy-sensitive relapsed DLBCL [59].

Consolidation Phase II trials showed mitigated results. Some studies suggest an interest for anti-CD20 RIT in untreated [57] or R/R patients [41], whereas others alerted researchers to the potential toxicities of such a regimen [58]. In MCL, it was suggested that radiolabeled anti-CD20 could be an effective therapeutic strategy for untreated [24,25,26] or R/R patients [29]. In DLBCL, a pilot study suggested that tandem consolidation therapy using ^90^Y-ibritumomab tiuxetan followed by HDT with Auto-SCT is not feasible for the treatment of high-risk patients with DLBCL who are in remission after R-CHOP, and the pilot study failed to provide any beneficial effects [31]. Conversely, a recent study showed interesting response rates for relapsed DLBCL patients, especially in terms of long-term response as well as an acceptable safety profile [32].

#### 2.1.4. Other Hematologic Diseases

In pathologies other than B-NHL, Phase I anti-CD20 RIT showed good outcomes and tolerance for a conditioning regimen followed by auto-SCT for MM (ORR: 73% and CR: 23%) [45] or for R/R Hodgkin lymphomas (2 CR/12 patients) [64]. RIT for hematologic malignancies mainly rely on anti-CD20 approaches. However, other strategies have been explored and have demonstrated interesting results, e.g., with Y-90 anti-CD22 RIT in R/R ALL [65], in DLBCL [66], and in B-NHL [67] with Bi-213 anti-CD33 in AML [68], and for conditioning with Re-188 or Y-90 anti-CD66 for AML [69] or pediatric hematologic diseases [70].

#### 2.1.5. Modest Efficacy?

Even though the clinical interest and good RIT tolerance were undeniable for some hematologic malignancies such as FL, which has led to the approval of the two main radiolabeled anti-CD20 mAb, the last decade failed to extend the use of RIT in other indications. Indeed, multiple promising Phase II clinical trials have explored RIT in front-line consolidation or conditioning strategies. Furthermore, the rare subsequent Phase III trials failed to highlight a significant difference in anti-CD20 RIT compared to cold rituximab. Because it is well-tolerated globally, anti-CD20 RIT might be of interest for specific fragile populations such as the elderly. However, other modalities of anti-CD20 RIT, such as multi-fractionation, should be explored to determine an optimal RIT regimen for aggressive B-NHL, as their therapeutic response differs from indolent B-NHL [49]. Otherwise, RIT also faces major drawbacks, such as the economic discontinuation of ^131^I-tositumomab in 2014, ^90^Y-ibritumomab tiuxetan supply difficulties, some complex practical limitations due to the need of specialized centers, the cost of treatment, and the emergence of new treatments such as ibrutinib, which highly restrict the expansion of RIT in hematologic malignancies.

### 2.2. RIT for Solid Tumors: A Viable Strategy?

RIT of solid tumors represents a smaller part among the total number of studies that were identified (around 35% of the total) (Figure 2). In this section, we will first discuss RIT performed with full mAbs as the targeting vectors and then those investigating antibody fragments, such as F(ab′)_2_, Fab′, or ScFv. Finally, alternative RIT strategies, such as the two-step pretargeting approach, will also be described to investigate their advantage in terms of efficacy compared to the radiolabeled mAbs.

#### 2.2.1. Overview of the Last Ten Years of RIT Involving Full-Length Antibodies

Despite improvements in radiochemistry processes and RIT protocols, the expansion of RIT for solid tumors has remained quite limited. In addition, there are two-times less clinical trials with radiolabeled full mAbs (phases I to III) for solid tumors that have been conducted over the last ten years, other than those reported by Pouget et al. in 2011 (20 vs. 48) (Table 3). We observed a lack of Phase III trials among all of the published RIT clinical trials with full mAbs as vectors, with ~50% being Phase I trials, ~40% being Phase I /II assessments, and two studies being retrospective or unspecified. Most of them assessed feasibility and toxicity, thereby making it complicated to clearly determine the therapeutic benefits of RIT. Table 2 also indicates the administration routes other than intravenous injection used in the clinical studies. Clinical trials involving local RIT injections demonstrated an absence of significant hematotoxicity and favored a higher dose delivered in situ. In addition, a retrospective study showed a dose delivered to the cerebral spinal fluid of 32.1 Gy in patients after the intraventricular injection of ^131^I-radiolabeled mAbs targeting GD2 or B7H3, which is neither associated with radionecrosis (<1%) nor neurologic defects [71]. With those encouraging results, intracerebroventricular RIT is currently being assessed in new clinical trials involving patients with central nervous system tumors (NCT 03275402, NCT 04022213, NCT04315246, NCT04167618, NCT03276572, and NCT04743661/EudraCT 2020-000670-22). Most of the clinical trials reported for RIT on solid tumors involved a limited number of patients who were already in the metastatic phase and who were resistant to first-line or second-line treatments. We can however discuss the target, mAb or radionuclide, as well as the addition of cold mAb or its combination with an active molecule such as chemotherapy.

#### 2.2.2. Combination RIT

In contrast to RIT for hematologic tumors, 63% of the clinical studies conducted on solid tumors were not associated with any other additional treatment or cold antibody. Some trials used Omburtamab, Girentixumab, J591, Labetuzumab, (the majority being IgG_1_), which possess antibody-dependent cell-mediated cytotoxicity (ADCC), complement-dependent cytotoxicity (CDC), and/or antibody-dependent cell-mediated phagocytosis (ADCP) mechanisms, but a only few of the studies investigated the association of RIT and the cold antibody [72]. Among them, two studies, based on trastuzumab reported the concomitant administration of cold mAb during RIT protocols [73,74]. 

**Table 3:** Overview of RIT of solid tumors in clinical trials, from 2010 to 2021.

Target/Vector	Isotope	Clinical Phase ^1^	*n ^2^*	Association ^3^	Cold mAb (+/−)	Fractionation (+/−)	PFS (Months) or X-Year PFS (%)	OS (Months) or X-Year OS (%)	NCT	Year of Publication	Ref
HER2-expressing breast cancer, peritoneal carcinomatosis or gastric cancer											
HER2/Trastuzumab	Pb-212	I	16	−	+	−	−	−	−	2014	[73]
	Lu-177	I	10	−	+	−	−	−	−	2017	[74]
Medulloblastoma and neuroblastoma											
B7H3/Omburtamab	I-131	Retro	94	(R)	−	+	−	−	NCT00445965 NCT00089245	2015	[71]
GD2 /3F8		II	43	-	−	+	11	24.9	−	2018	[75]
Metastatic colorectal cancer											
A33/huA33	I-131	I	19	(C)	−	−	5	28.7	NCT00291486	2014	[76]
CEA/cT84.66	Y-90	I/II	16	(C)	−	−	9.6	41.2	−	2017	[77]
CEA/Labetuzumab	I-131	II	63	-	−	−	16	55	NCT27763687	2017	[78]
Metastatic melanoma											
MSCP/cDTPA-9.2.27	Bi-213	I	38	-	−	+	20.4	−	−	2011	[79]
Metastatic pancreatic cancer											
hPAM4 (MUC-1)/Clivatuzumab tetraxetan	Y-90	I	21	-	−	+	1.3	4.3	NCT00603863	2011	[80]
		I	42	(C)	−	+	−	7.7	NCT01956812	2012	[81]
		Ib	58	(C)	−	+	−	7.9 (A) vs. 3.4 (B)	NCT01956812	2015	[82]
Metastatic prostate cancer											
PSMA/J591	Lu-177	II	47	-	+	−	−	22.2 vs. 11.4	NCT00195039	2013	[83]
		I/II	49	-	−	−	−	42.3	NCT00538668	2019	[84]
		I	15	(C)	−	−	−	−	NCT00916123	2020	[85]
		I/II	6	-	−	+	−	−	NCT00538668	2020	[86]
Metastatic renal cell carcinomaz											
CAIX/Girentuximab	Lu-177	I	23	-	−	+	11.1	25.3	−	2013	[87]
		II	14	(C + R)	−	+	−	−	NCT02002312	2016	[88]
Non-small cell lung cancer											
DNA/chTNT	I-131		96	(C + R+Pmc)	−	−	−	23–29.1	−	2016	[89]
Sarcoma											
FZD10/OTSA-101	Y-90	I	20	-	−	+	PR in 3/8	−	NCT01469975	2018	[90]
B7H3/Omburtamab	I-131	I/II	52	-	−	−	−	−	NCT01099644	2020	[91]

^1^ Retro: retrospective. ^2^ *n*: number of patients. ^3^ (C): chemotherapy; (R): external-beam radiotherapy; Pmc: percutaneous microwave coagulation therapy. PFS: progression-free survival; OS: overall survival.

However, both were pilot trials that were only assessing the radiolabeling and radiotoxicity of the radiolabeled trastuzumab. Interestingly, targeting PSMA with a mixture of ^177^Lu-J591 and cold J591 mAbs was associated with a decrease in the circulating tumor cells in 82% of patients (14 out of 17) bearing detectable circulating tumor cells [84]. It has also been recently demonstrated that the co-administration of unconjugated mAb with its cognate antibody–dye conjugate increases its penetration into solid tumors [92]. The impact of this antibody concentration on radiolabeled antibody biodistribution remains undemonstrated and should be confirmed—if possible—at the microscopic level. Another study related a synergistic association of the antibody hu33, an antibody directed towards the A33 antigen, and chemotherapy (gemcitabine) in patients with CRC [76]. No other combination RIT has been reported in previously clinical trials, thereby highlighting that idea that RIT was expected to be effective alone.

#### 2.2.3. Choice of the Targets/mAbs/Radionuclides

If RIT of hematologic cancers has focused on four different antigen targets (i.e., CD20, CD22, CD33 and CD66) until now we have identified 12 different antigens for RIT of solid tumors, with each one only being assessed once or twice in clinical trials. The multiplication of targets makes it difficult to compare different studies to determine whether RIT is effective. Considering CRC RIT, the A33 target is of more interest than the canonic CEA target. Indeed, this protein is retained on the membrane and is not released in the blood, thereby limiting hematotoxicity and enhancing bioavailability [77,78]. Alternatively, targeting antigens on circulating tumor cells (CTC) is of interest for metastatic cancers, as demonstrated with RIT using radiolabeled J591, which is associated with a CTC decrease [84]. One of the reasons why it is difficult for RIT to be successful in the treatment of solid tumors is because it relies on the penetration of full mAbs and the subsequent low dose that is delivered. Tumor vasculature is indeed abnormal, with the leaky vessels and the presence of a high complex extracellular matrix impeding tumor cell access to mAbs. Furthermore, hypoxia, which is present in most of solid tumors, reduces radiation efficiency, except for that of alpha particles, which do not need oxygen. Solid tumor features that modulate RIT efficiency were clearly described in a recent review [93]. To circumvent these limitations, one solution could be to perform repeated treatment cycles to limit radiotoxicity. This approach was tested in different trials, with substantial results in patients with metastatic renal cell carcinoma [88], neuroblastoma or medulloblastoma [71], melanomas [79], pancreatic cancer [81,82], or sarcomas [90,91] (Table 3). It is important to note that in patients with mCRPC, the targeting of PSMA with ^177^Lu-J591 through fractionated doses did not provide any benefits [86]. A recent study reported the association of ^177^Lu-J591 and docetaxel/prednisone in a cohort of 15 patients with mCRPC without inducing additional toxicity, with taxanes being assumed to be radio sensitizers [85]. It is well known that PSMA is highly specific to prostate, cancer and further clinical trials will thereby consider the efficacy of J591 radiolabeled with the highly potent Ac-225, which gives rise to alpha particles (NCT03276572). Alpha particles (e.g., Pb-212 or Bi-213) have thereby been evaluated in two clinical trials (either on melanoma or peritoneal carcinomatosis from HER2+ primary lesions). These radionuclides-generating alpha particles with a high LET have a very short half-life and do not induce any radiotoxicity [73,79]. One problem of this very attractive strategy is the difficulty of acquiring radionuclides [94]. For instance, the production of Actinium-225 in 2018 would have only supported the treatment for several hundred patients [93].

## 3. Alternatives to Conventional RIT and Prospects

IgGs are excellent vectors for carrying a payload towards a membrane antigen. However, their size (~150 kDa) impedes their filtration via the kidneys—which possess a glomeruli threshold at around 70 kDa—thus resulting in major hepatocellular uptake. In addition, the Fc region of IgGs contains a FcRn sequence (i.e., neonatal Fc receptor, or Brambell receptor), a heterodimer derived from the major histocompatibility complex class I receptor that is involved in folding, transport, and FcRn functions [95]. The ability of the Fc region of IgG to bind FcRn protects them from lysosomal degradation and induces their translocation back to the cell surface. While IgG recycling is highly dependent on the IgG subclass, it still results in a serum half-life of 7 to 21 days (for IgG_3_ or IgG_1,2,4_, respectively). The long half-life of IgGs represents a major drawback underlying RIT of solid tumors, as it causes hematotoxicity and resistance phenomena due to the slow penetration of mAbs into the center of the tumor [3]. Thereby, two main strategies have been explored to circumvent these issues, both of which are based on improving the pharmacokinetic of the vector carrying the radionuclide. Firstly, the antibody fragments have been considered as a vector instead of as full-sized IgGs; secondly, RIT can be performed in two steps using pretargeting approaches.

### 3.1. Fragments-Based RIT

**Figure 3:**
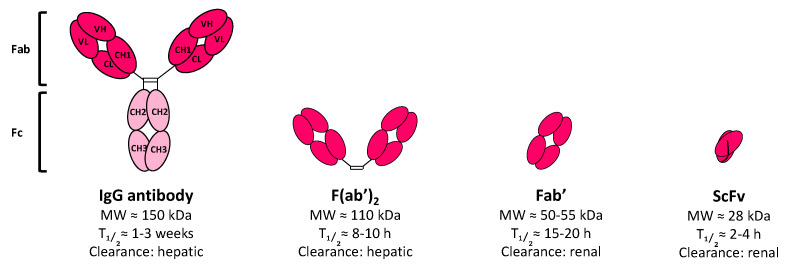
IgG fragment formats assessed in clinic for RIT. The molecular weight (MW), blood half-life (T12), and main clearance route are given for each format.

IgG fragments possess a smaller size, resulting in faster biodistribution (Figure 3). Their characteristics promote penetration in tissue as well as quicker clearance from the blood via the kidneys, and these properties are very useful for a wide variety of diagnosis or therapeutic applications [96,97,98]. In addition, fragments lack an Fc domain, thereby impeding both FcRn recycling and immune response-mediated Fc (i.e., CDC and ADCC). The main antibody fragments that have been assessed for oncology applications are schematized in Figure 3. Among them, antigen-binding antibody fragments, such as F(ab′)_2_ or Fab′ can be produced directly via the proteolytic cleavage of natural antibodies or can be designed by genetic engineering methods, while single chain variable fragments (ScFv) are only genetically engineered. Fragments are low costs compared to full mAbs because they can be easily produced in microbial, yeast, or human cells. All of them are designed to retain at least the same antigen specificity as full-length mAbs and can be used either as diagnosis tools or to carry a therapeutic payload to the tumor target.

#### 3.1.1. Fab′

Fab′ is the oldest fragment to be assessed in clinic, with its first FDA approval being received in 1994 to prevent blood clot formation in angioplasty (Abciximab, ReoPro®, Centocor/Eli Lilly) [98]. To date, several Fab′ fragments have been also approved by the US FDA, EMA, and Chinese State FDA agencies for a wide range of applications, including for the treatment of cancers (e.g., Ranibizumab, Lucentis® derived from Bevacizumab, Avastin®, both anti-VEGF) [96]. However, none have been approved for RIT as of yet. A divalent Fab′ maleimide fragment derived from the humanized A5B7 antibody radiolabeled with I-131 was assessed for RIT in 2002 in patients bearing CRC tumors in a very small cohort of pilot study [99]. The imaging follow-up demonstrated a specific signal in patients bearing CEA-expressing tumors. However, the quantification of the scan failed to demonstrate a significant uptake in tumors compared to in the other organs. Moreover, MIRD assessment dosimetry was unfavorable for the divalent Fab′ maleimide due to a high uptake in the kidneys. Aggregates were also observed for three patients (of a total of ten) at later time points (up to 29% at 24 h), which was correlated to the amount of CEA antigens measured in patients’ serum [99].

Aggregation is one of the most well-known major drawbacks of antibody fragments, which can vary according to fragment type [100]. While the feasibility was clearly demonstrated, to the best of our knowledge, no other clinical assessments involving this Fab′ fragment have been conducted in the last ten years (Table 4).

It is interesting to note that Fab′ fragments have however demonstrated some potential for diagnosis. For example, the ^99m^Tc-nofetumimab merpentan Fab′ fragment (Verluma®, Boehringer Ingelheim Pharma KG, Ingelheim, Germany) targeting the pancarcinoma antigen expressed in cancer cells has been FDA-approved in 1992 for the diagnosis of small-cell lung cancer but was discontinued in 2013 [108]. The ^99m^Tc-sulesomab Fab′ (LeukoScan®, Immunomedics, Morris Plains, NJ, USA) received EMA-approval in 1997 for the SPECT imaging of activated granulocytes in patients with osteomyelitis, but it was also discontinued in 2018 [109].

#### 3.1.2. F(ab′)_2_

In 2011, the centruroides (scorpion) immune F(ab′)_2_ (equine) (Anascorp®, Rare Disease Therapeutics, Inc., Franklin, TN, USA) has been the first F(ab′)_2_ to be FDA-approved, for the treatment of clinical signs of scorpion envenomation [110]. This was followed by the FDA-approval of crotalidae immune F(ab′)_2_ (equine) (Anavip®, Rare Disease Therapeutics, Inc., Franklin, TN, USA) in 2015 for the management of coagulopathic effects in adults or in pediatric patients with North American Pit Viper envenomation (from cottonmouth, copperhead or rattlesnake) [111]. The phase III clinical assessments demonstrated a similar safety but also a better efficacy in treating venom-caused hematologic toxicity and a higher stability over time (3 years) than the Fab′ antivenom version that was approved in 2007 (CroFab®, BTG, UK). However, in oncology, the efficacy of F(ab′)_2_ is more contrasted. Concerning RIT, F(ab′)_2_ has been the most common fragment type assessed in clinical trials and still represent five studies out of the seven conducted over the last ten years that are listed in Table 4. Different antigens have been evaluated as potential targets for RIT with F(ab′)_2_ fragments [3], but only two have remained under investigation since 2010, CD147 [112] for metastatic solid cancers and tenascin C [101] for Hodgkin lymphoma. With the exception of CD147/metuximab, the targeting of tenascin C and CEA are still at early stage clinical assessments (Phase I/II) and only involve a small number of patients. In most cases, radiolabeling has been achieved with the conventional I-131 β-emitter (8 day half-life, mean LET of 0.25 keV/µm).

Several of the clinical trials listed in Table 4 have been performed with the ^131^I-metuximab HAb18G/CD147 F(ab′)_2_ fragment (Licartin®, Chengdu Hoist Hitech Co. Ltd, Chengdu, China), as a post-surgery treatment, or it has been combined with radiofrequency ablation in patients with recurrent metastatic hepatocellular carcinoma (HCC) [103,104,105,106,107,112,113]. RIT with metuximab was associated with mild to moderate hematologic/hepatic toxicities, ≤Grade 3, especially for injected activities ≥27.75 MBq/kg; however, this amount of activity is considered to be the most appropriate amount in order to obtain therapeutic efficacy in most studies. In addition, no damages in thyroid function nor significant positive human anti-murine antibody (HAMA) response were observed in most of the patients after two cycles of treatment. The median OS was found between 20 to 60 months for the most recent study, which was associated with a 5-year PFS of around 43.4% for the treated group compared to 21.7% for the control group [107]. In April 2015, Licartin® ^131^I-metuximab HAb18G/CD147 became the first radioconjugates F(ab′)_2_ to receive approval by the China State FDA (n°S20050039) [113,114].

While fragments have mainly been assessed for RIT of solid tumors, one phase I/II study in 2014 reported the feasibility of such an approach to treat chemorefractory Hodgkin lymphoma [101]. ^131^I-tenarad F16SIP, an 80-kDa F(ab′)_2_, recognizes the extra-domain A1 of tenascin C, which is highly expressed in extracellular matrix of tumors. Eight patients received 2.05 GBq/m^2^ after a median of three previous lines of chemotherapy, and seven out of the eight patients had undergone bone marrow transplantation. All of the patients showed moderate to severe thrombocytopenia (up to Grade 3) plus Grade 2/3 neutropenia and lymphopenia, and one patient suffered from severe multilineage hematological toxicity (Grade 4) 5 weeks after the administration of the first dose of Tenarad (4.18 GBq). While this single study demonstrated the proof-of-concept of tenascin C targeting for RIT, the high related toxicity implies mandatory protocol modifications for the administration of the most effective and the safest therapeutic option.

Despite encouraging clinical trials, RIT with the F(ab′)_2_ fragment is still not approved by the US and European agencies. Furthermore, to the best of our knowledge, there is no F(ab′)_2_ that has been approved by the US FDA or EMA for use in oncology.

#### 3.1.3. ScFv

Single-chain antibody fragments (ScFv) are genetically engineered fragments of about 28 kDa and are composed of the antibody V_H_ and V_L_ domains connected by a flexible polypeptide linker (Figure 3). Their small size allows for their quick clearance via kidney glomeruli filtration after a few hours. In oncology, ScFv fragments have been widely used in chimeric antigen receptor (CAR) T-cell-based immunotherapy to engineer T-cells with a recombinant receptor in order to redirect them toward tumor-associated antigens [115]. In 2017/2018, two CD19-targeted CAR T-cells were approved by the US FDA for the treatment of hematologic tumors: R/R B-cell ALL and/or R/R DLBCL (Tisagenlecleucel, Kymriah®, Novartis, Cambridge, MA, USA; Axicabtagene ciloleucel, Yescarta®, Kite Pharma, Los Angeles, CA, US) [115]. Moreover, ScFv possessing an antigen-binding capacity have also been used for the engineering of natural killer (NK) cell immune checkpoint inhibitors to improve their anti-cancer activity [116]. However, in both cases, the ScFv is used for ex vivo engineering construction and is not administered alone in patients as a therapeutic protein. To the best of our knowledge, the only FDA approval for ScFv antibody fragments has concerned applications outside of the oncology field, such as Brolucizumab (Beovu®, Novartis, Cambridge, MA, USA), a humanized 26-kDa ScFv inhibiting VEGF-A that was US FDA-approved in October 2019 for the treatment of neovascular (wet) age-related macular degeneration [117].

In RIT clinical trials (Table 4), the only study describing the use of radiolabeled ScFv was performed in 2011 through a phase I trial using ^131^I-CIGB-M3 trivalent ScFv that is specific for CEA targeting in patients bearing colorectal metastasis [102]. The feasibility was assessed on a small cohort of 17 patients who were divided into two groups that received either 0.3 mg or 1 mg of CIGB-M3 ScFv for similar injected activities of about 185–259 MBq of I-131. Toxicity evaluations demonstrated the low off-target toxicity of the ScFv fragment in both groups and was associated with lower HAMA responses compared to patients receiving a single 1 mg dose of the parental CB-CEA-1 full antibody. While trivalent CIGB-M3 ScFv demonstrated interesting pharmacokinetic outcomes and quite favorable dosimetry (0.07 ± 0.02 to 0.08 ± 0.02 mGy/MBq in the whole body, 1.1 ± 0.6 to 2.0 ± 1.3 mGy/MBq in kidneys, it has however not been pursued clinical development so far.

#### 3.1.4. Limitations and Prospects of Antibody Fragments

The investigations summarized in Table 4 illustrate the difficulty of engineering robust antibody fragments with high affinity towards antigens, favorable pharmacokinetic profiles, low toxicity, and good stability over time. Fragments must also be able to undergo harsh radiochemical processes without the induction of deleterious effects on the previously mentioned properties. Concerning small antibody fragments, such as ScFv or single-domain antibodies, which are also called nanobodies, 15-kDa fragments derived from Camelidae antibodies, radiochemical processes are known to dramatically affect their affinity towards antigens [98,118]. These small fragments are also more prone to aggregation and possess a lower thermostability than full IgGs or bigger fragments. The scare and high disparity of clinical studies also makes it difficult to conclude about the efficiency of antibody fragments for RIT application. Indeed, the low number of studies, the low number of enrolled patients, and the high variety of fragment formats or targets is quite disappointing, resulting in little evidence representing the efficacy of RIT with fragments. However, such strategies remain of interest for RIT or nuclear imaging applications, but these strategies still require optimization. Recent advances in phage-display technology, chemical and chemoenzymatic engineering, and radiolabeling protocols have introduced new insights to circumvent most of these drawbacks and have shown promising outcomes for antibody fragments in preclinical studies [5,119].

### 3.2. Pretargeted Radioimmunotherapy (PRIT)

**Figure 4:**
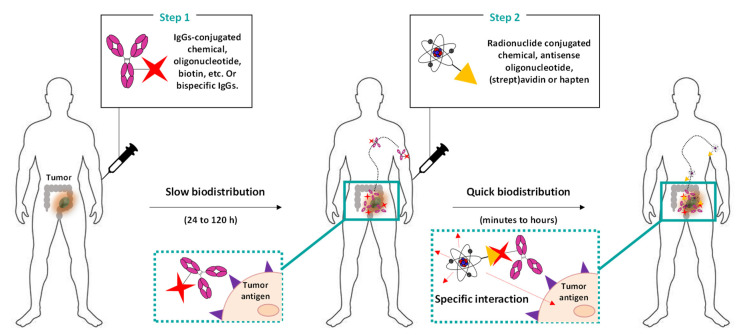
Pretargeted radioimmunotherapy (PRIT): general strategy. Antibodies or fragments are first functionalized with biotin, avidin, and oligonucleotides (e.g., phosphorodiamidate morpholinos, peptide nucleic acids) and are then chemically engineered to be bispecific. Then, the conjugated mAbs are administered (in most cases intravenously). After a delay of 24–72 h (to allow for the sufficient clearance of unbounded conjugated mAbs), radionuclides functionalized with a specific counterpart that is only able to recognize the conjugates attached to the mAbs (such as biotin, (strept)avidin, countersense oligonucleotide, chemical of haptens) are administered (intravenously, intraperitoneally, or locally). Due to the small size of the radioligand, the biodistribution and clearance are very quick (few hours), thereby limiting the off-target irradiation of healthy tissues.

The other potential strategy for improving clearance and to reduce off-target toxicity is to delay the administration of the radionuclide from one of the mAbs, delaying administration by a few hours to several days, through the so-called pretargeting (or PRIT) approach (Figure 4) [120,121].

Full mAbs or fragments are conjugated with a biological or chemical moiety and are first administered to have time to accumulate into the tumor before the administration of the radioactive component (delayed from few hours to several days) [121]. The radioligand is designed to carry a reactive payload that is highly specific to one of the conjugated mAbs. In addition, a high affinity is required between the two reactive species in order to obtain a quick reaction (*k*_2_ = 10^1^–10^7^ M^−1^s^−1^). Due to the small size of the radioligand, there is a quick biodistribution and a short half-life in the blood. The PRIT approach is thereby particularly interesting to reduce the off-target toxicity of heathy tissues.

Different PRIT strategies have been developed in the last 30 years. The oldest strategy involves a very high affinity between the biotin and (strept)avidin proteins. Despite interesting outcomes in preclinical trials, clinical trials phase I/II demonstrated the important immunogenicity of (strept)avidin and the off-target binding of endogenous biotin. Regarding those drawbacks, biotin-(strep)avidin pretargeting systems have still not yet been introduced to the market. Another PRIT strategy based on bispecific (or multispecific) antibodies (bsAbs) or fragments was developed soon after. This strategy required genetic and chemical engineering to design bsAbs with one (or two) antigen binding domain directed against a tumor antigen plus another one that was specific to a small, radiolabeled peptide. The engineering process led to the development and clinical trials of several bsAbs formats for oncology; their advantages/drawbacks and applications are detailed in specific reviews such as [122,123]. To date, only three bispecific formats have been marketed: (i) blinatumomab (Blincyto®, Amgen, Thousand Oaks, CA, USA), a bispecific tandem ScFv anti-CD3 × CD19 that became FDA-approved in 2015 for the treatment of ALL and B-ALL; (ii) emicizumab (Hemlibra®, Roche, Basel, Switzerland), a bispecific IgG_4_ anti-FIX × FX that was EMA-approved in 2018 for the restoration of the missing FVIIIa function in patients with haemophilia A [124]; and (iii) catumaxomab (Removab®, Trion Pharma GmbH, Munich, Germany), a chimeric bispecific rat/mouse IgG_1_ anti-EpCAM × anti-CD3 that became EMA-approved in 2009 for the treatment of patients with malignant ascites and that was withdrawn for economic reasons in 2013 (US) and 2017 (EU).

In PRIT clinical assays, bsAbs have been developed to target CEA (Table 5). In 2012, a phase II study was performed with a chimeric bispecific human/mouse (hMN-14 × m734) F(ab′)_2_, an anti-CEA × anti-diethylenetriaminepentaacetic acid (DTPA) radiolabeled with I-131 for the treatment of patients with medullary thyroid carcinoma [125]. The administration of bsAbs (40 mg/m^2^) and ^131^I-DTPA (1.8 GBq/m^2^) were delayed from 4 to 6 days. The results showed that PRIT was well tolerated in most of the patients and that it was associated with low increases of HAMA and human anti-human antibody (HAHA) responses (2.3% and 26.2%, respectively). In addition, the efficacy of PRIT was demonstrated to have disease control in 76% of patients and was associated with a median PFS of 13.6 months and a median OS of 43.9 months. Recently, the chimeric (hMN-14 × m734) F(ab′)_2_ has also demonstrated interesting outcomes for the treatment of metastatic colorectal cancer [78].

A new generation of bsAbs formats have then been assessed to decrease the observed immunogenicity of the chimeric F(ab′)_2_. Trispecific humanized TF2 Fab′ is composed of an anti- histamine-succinyl-glutamine (HSG) fragment that is derived from the 679 anti-HSG IgG_1_ and 2 fragments of the humanized anti-CEA derived from hMN-14 IgG_1_ (labetuzumab®, CEA-CIDE, Immunomedics, Inc., Morris Plains, NJ, USA) [128]. The first-in-man study of Schoffelen et al. (NCT00860860), which was published in 2013, demonstrated the feasibility and safety of PRIT with TF2 and ^177^Lu-IMP-288 (a 1.5 kDa HSG peptide) in patients bearing metastatic CRC [126]. A previous imaging study with TF2/^111^In-IMP-288 determined that an administration interval of 24 h between the administration of both treatments was the most suitable. Patients received a mean injected activity of 5.6 GBq (dose escalation from 2.5 to 7.4 GBq), with no significant differences being observed between cohorts. However, the HAHA response toward TF2 was measured to be quite high (mean 386 ng/mL^−1^) in 11 of the 21 patients 1 week following the administration, and this response gradually increased over the 8-week follow-up period, suggesting that TF2 was surprisingly immunogenic despite being humanized and lacking an Fc portion. PRIT with bsAbs thereby represents a viable alternative for metastatic cancers but still requires further study to optimize the appropriate medication and to reduce immunogenicity.

More recent pretargeting systems involving oligonucleotides or chemical moieties have demonstrated promising outcomes in preclinical studies. While the low stability of oligonucleotides still impedes its clinical transfer, systems employing bioorthogonal chemistry only entered clinical trials testing their application as antibody-drug conjugate (NCT04106492) last October. In such strategies, mAbs are functionalized with the *trans*-cyclooctene (TCO), and the radioligand is conjugated to tetrazines (Tz), with both entities being non-immunogenic and highly reactive (*k*_2_ up to 10^6^ m^’1^s^’1^) and able to specifically interact in vivo to form a covalent bond through the inverse-electron demand [4 + 2] Diels–Alder cycloaddition [121]. The application of the TCO/Tz bioorthogonal reaction is however still challenging, especially when considering the isomerization risks of the TCO ring, which is photosensitive but that still represents a potential prospect for the PRIT of solid tumors.

## 4. Conclusions

Despite numerous clinical trials assessing the FDA-authorized anti-CD20 radiopharmaceuticals ^90^Y-ibritumomab-tiuxetan and ^131^I-tositumumab through different strategies (first-line, consolidation, SCT-conditioning), the last ten years have failed to expand anti-CD20 RIT indications but have confirmed that RIT using radiolabeled anti-CD20 remains a pertinent choice for patients with follicular lymphomas, especially after relapse. These radiolabeled mAbs can indeed enhance survival in low-grade B-NHL. However, even though the use of the cold anti-CD20 mAb, rituximab, showed excellent results when applied generally, it may have limited the use of RIT in hematologic malignancies. In contrast to the last decade, there are, to our knowledge, only 10 ongoing clinical trials of RIT for hematologic malignancies, and among them, only one targets CD20 (Table 6). This may traduce a decline of interest for this therapeutic strategy in hematologic malignancies.

Moreover, in solid tumors, there is no RIT that has been validated by the FDA nor the EMA as of yet. Metuximab only received China State FDA approval in 2005, and the data from clinical trials are quite disappointing. Despite encouraging results highlighting the interest of several different potential targets, there are still few data available, and most of the studies did not reach the phase III investigation step. However, it is interesting to note that the amount of RIT in solid tumors has increased significantly and has recently reached a peak, thereby representing more than 50% of the on-going RIT clinical trials (Table 6). The high variety of newly discovered targets/antibodies combined with the use of potent α-emitters suggests new promising prospects for RIT in solid tumors, especially for metastatic malignancies. In addition, recent advances in small mAbs fragment bioconjugation as well as pretargeted strategies represent assets for improving the efficacy and for expanding RIT for solid tumors in the next decade.

## Figures and Tables

**Table 1:** Overview of anti-CD20 RIT for non-solid cancers in clinical trials from 2010 to 2021. (For details, see Supplementary Table S1).

Target/	Isotope	Cancer Type ^1^	*n* ^2^	Type of Studies					Line of Treatment				[Ref]
Vector				I	I/II	II	III	Others ^3^	1st Line	Conditioning	Consolidation	R/R ^4^	
*Ibritumomab* *tiuxetan*	Y-90	FL	12	1	1	9	1	−	4	1	6	1	[10,11,12,13,14,15,16,17,18,19,20,21]
		MZL	4	−	−	3	−	1 (P)	2	−	1	1	[13,17,22,23]
		MCL	7	1	−	4	−	1(R)/1(P)	−	1	5	1	[24,25,26,27,28,29,30]
		DLBCL	4	−	−	3	−	1(R)	−	2	2	−	[31,32,33,34]
		Burkitt Lymphoma	1	−	−	−	−	1(R)	−	−	1	−	[35]
		B-NHL	9	2	−	5	−	1(R)/1(CS)	−	5	1	3	[36,37,38,39,40,41,42,43,44]
		MM	1	1	−	−	−	−	−	1	−	−	[45]
*Rituximab*	I-131	FL	1	−	−	1	−	−	1	−	−	−	[46]
		B-NHL	3	−	1	1	−	1(R)	−	1	−	2	[47,48,49]
	Lu-177	Low-grade B-NHL	1	−	1	−	−	−	−	−	−	1	[50]
		B-NHL	1	−	−	−	−	1(P)	−	−	−	1	[51]
	Y-90	B-NHL	1	−	−	−	−	1(CS)	−	−	−	1	[52]
*Tositumomab*	I-131	FL	2	−	−	−	2	−	−	−	2	−	[53,54]
		Low-grade B-NHL	2	−	−	1	1	−	−	−	−	2	[55,56]
		DLBCL	3	−	−	2	1	−	−	1	2	−	[57,58,59]
		B-NHL	2	2	−	1	−	1(CS)		2	−	2	[60,61,62,63]
		HL	1	1	−	−	−	−	−	−	−	1	[64]

^1^ B-NHL: B-cell non-Hodgkin lymphoma; DLBCL: diffuse large B-cell lymphoma; FL: follicular lymphoma; HL: Hodgkin lymphoma; MCL: mantle cell lymphoma; MM: multiple myeloma; MZL: marginal zone lymphoma. ^2^ *n*: number of studies. ^3^ CS: case series; P: prospective; R: retrospective. ^4^ R/R: relapsed/refractor.

**Table 2:** Overview of other RIT approaches for non-solid cancers in clinical trials from 2010 to 2021.

Target/ Vector	Isotope	Cancer Type ^1^	Phase	*n ^2^*	Line of Treatment ^3^	Association ^4^ (+/−)	SCT (Auto/Allo/−)	Cold mAb (+/−)	Fraction-Ation * (+/−)	PFS Median (Months) or *X Years PF* ^5^ *(%)*	OS Median (Months) or *X Years OS (%)*	ORR (%)	CR (%)	NCT (or eq.), [Ref]
CD22														
*Epratuzumab tetraxetan*	Y-90	ALL	I	17	R/R	−	−	+	−	6.1	3.6	−	−	NCT01354457, [65]
		DLBCL	II	71	Consolidation	+ (C)	−	+ (anti-CD20 mAb)	−	NR *2 y: 82%*	NR *2 y: 89%*	−	77	NCT00906841, [66]
		B-NHL	I	18	R/R	−	−	+ (anti-CD20 mAb)	−	6.2	−	53	18	- [67]
CD33														
*Lintuzumab*	Bi-213	AML	I/II	31	1^st^ line or R/R	+ (C)	−	−	+	−	4.6	19	10	- [68]
CD66														
*Anti-CD66*	Re-188	AML	II	58	Conditioning	+ (C+I)	Allo	−	−	*2 y DFS: 38%*	*2 y: 38%*	−	−	- [69]
*BW250-183*	Y-90	Pediatric malignant (Mal.) and non-maligant (Non-Mal.) hemopathies	II	30	Conditioning	+ (C)	Auto Allo	−	−	Mal.: 12 Non-Mal.: NR	Mal.: NR Non-Mal.: NR	−	−	- [70]

^1^ ALL: Acute lymphoblastic leukemia; AML: acute myeloid leukemia; B-NHL: B-cell non-Hodgkin lymphoma; DLBCL: diffuse large B-cell lymphoma. ^2^ *n*: number of patients. ^3^ R/R: relapsed/refractory; SCT: stem cell transplantation. ^4^ (C): chemotherapy; (I): immunotherapy. ^5^ CR: complete response; DFS: disease-free survival; NR: non-reached; ORR: overall response rate; OS: overall survival. * Fractionation concerns RIT protocol.

**Table 4:** Overview of fragment-based RIT in clinical trials.

Target	Vector	*n* ^1^	Phase	Isotope	Application ^2^	Year of Publication	NCT or eq.	Ref
Non-solid cancer								
Tenascin C	F16SIP F(ab′)_2_	8	I/II	I-131	HL	2014	EudraCT2007-007259-15	[101]
Solid cancers								
CEA	CIGB-M3 ScFv	17	I	I-131	CRC *	2011	–	[102]
CD147	HAb18 metuximab F(ab′)_2_ (Licartin®)	110	III	I-131	HCC *	2010	NCT00829465	[103]
		68	P	I-131	HCC *	2012	ChiCTR-TRC-08000250	[104]
		60	II	I-131	HCC *	2013	–	[105]
		127	III	I-131	HCC *	2014	ChiCTR-TRC-10000837	[106]
		156	II	I-131	HCC *	2020	NCT00819650	[107]

^1^*n*: number of patients. ^2^ CRC: colorectal cancer; HCC: hepatocellular carcinoma; HL: Hodgkin lymphoma. * Small metastases derived from those primary origins. P: prospective.

**Table 5:** Overview of PRIT in clinical trials over the last ten years.

Target	Antibody	Vector	*n*	Phase	Isotope	Application ^1^	Year of Publication	NCT	Ref
CEA	hMN14 × m734 F(ab′)_2_	Di-DTPA	42	II	I-131	MTC	2012	NCT00467506	[125]
			63	II	I-131	mCRC	2016	-	[78]
	TF2	IMP-288	21	I	Lu-177	mCRC	2013	NCT00860860	[126]
			18	I/II	Lu-177	SCLC and NSCLC	2015	NCT01221675, EudraCT 200800603096	[127]

^1^ MTC: medullary thyroid carcinoma; mCRC: metastatic colorectal cancer; NSCLC: non-small cell lung carcinoma.

**Table 6:** Ongoing RIT in clinical trials, either for solid or non-solid tumors.

NCT	Starting Date	Phase	Target	Vector	Isotope	Application ^1^	Administration Route ^2^	Association ^3^ (+/−)	Cold mAb (+/−)
Non-solid tumors									
NCT01796171	2012	I/II	CD37	Betalutin	Lu-177	NHL/FL	I.V	-	+
NCT02320292	2015	III	CD20	Ibritumomab tiuxetan	Y-90	FL	I.V	-	+
NCT04082286	2016	I	CD66	Anti-CD66	Y-90	AL/AML	I.V	+ (T)	-
NCT03128034	2017	I/II	CD45	BC8-B10	At-211	AML	I.V	+ (C + R)	-
NCT02658968	2017	I	CD37	Betalutin	Lu-177	DLBCL	I.V	-	+
NCT03806179	2018	I	CD37	Betalutin	Lu-177	NHL/FL	I.V	-	+
NCT04466475	2020	I	CD38	OK10-B10	At-211	MM	I.V	+ (C + T)	
NCT04083183	2020	I/II	CD45	BC8-B10	At-211	Non-mal. hemopathies	I.V	+ (C + T + R)	+
NCT04856215	2021	II	CD66	Anti-CD66	Y-90	Leukemia	I.V.	+ (T + R)	-
NCT04871607	2021	II	CD25	Basiliximab	Y-90	R/R HL	I.V.	+ (C + R + T)	+
Solid tumors									
NCT02454010	2015	I	CDH3	FF21101	Y-90	Adv. Solid T.	Not. Spec.	-	-
ChiCTR-IPR-17011206	2017	III	CD147	Metuximab *	I-131	HCC	I.V.	–	–
NCT03724747	2018	I	Mesothelin	BAY2315497	Th-227	mCRPC	Not Spec.	+ (H)	-
NCT03507452	2018	I		BAY2287411	Th-227	OC, Adv. Solid T.	Not Spec.	-	-
NCT03275402	2018	II/III	B7H3	Omburtamab	I-131	CNS meta.	I.C	-	-
NCT04022213	2019	II			I-131	SRCT/PC		+ (R)	-
NCT04644770	2020	I	hK2^1^	h11B6	Ac-225	mCRPC	I.V.	-	-
NCT04674722	2020	I	HER2	NM02 **	Re-188	Breast cancer	I.V.	-	-
NCT04315246	2020	I/II	B7H3	Omburtamab	Lu-177	LP meta.	I.V.	-	-
NCT04167618	2020	I/II			Lu-177	MB		-	-
NCT04743661 Eudra CT 2020-000670-22	2021	II			I-131	MB		+ (C)	-
NCT03276572	2021	I	PSMA	J591	Ac-225	mCRPC	I.V	-	-

^1^ Adv solid T.: advanced solid tumors; AL: acute lymphoma; AML: acute myeloid leukemia; CNS meta.: metastases from central nervous system tumors (neuroblastoma + leptomeningeal metastasis); DLBCL: diffuse large B-cell lymphoma; FL: follicular lymphoma; HCC: hepatocellular carcinoma; hK2: human kallikrein-2; LP meta.: leptomeningeal metastasis; MB: medulloblastoma; mCRPC: metastatic castration-resistant prostate cancer; MM: multiple myeloma; NHL: non-Hodgkin lymphoma; NSCLC: non-small cell lung carcinoma; Not Spec.: not specified; SLCL: small-cell lung cancer; OC: ovarian cancer; PC: peritoneal cancers; R/R HL: relapsed/refractory Hodgkin lymphoma; RSRCT: small-round cell tumors. ^2^ I.V.: intravenous; I.C.: intracerebroventricular. ^3^ (C): chemotherapy; (H): hormone therapy; (R): external-beam radiotherapy; (T): autologous hematopoietic stem cell transplantation. * F(ab′)_2_; ** single-domain antibody (nanobody).

## Supplementary Materials

The following are available online at https://www.mdpi.com/article/10.3390/cancers13215570/s1, Table S1. Details of anti-CD20 RIT for non-solid cancers in clinical trials, from 2010 to 2021.
