# Supplementary material for: Radioimmunotherapy in Oncology: Overview of the Last Decade Clinical Trials
Source: Cancers (Basel). 2021 Nov 7;13(21):5570. doi: 10.3390/cancers13215570 (PMC8583425; doi:10.3390/cancers13215570)
Supplement: Supplementary file 1 [file cancers-13-05570-s001.zip › cancers-1391265-supplementary.pdf]

cancers

Radioimmunotherapy in Oncology: Overview of the Last Decade Clinical Trials

Aurélie Rondon, Jacques Rouanet and Françoise Degoul

Table S1. Details of anti-CD20 RIT for non-solid cancers in clinical trials, from 2010 to 2021.

Target/ Vector	Isotope	Cancer type[1]	Phase[2]	*n*[3]	Line of treatment[4]	Association[5] (+/–)	SCT (Auto/ Allo/–)	Cold mAb (+/–)	Fractionation* (+/–)	PFS median (months) or *X-years PF*[6] *(%)*	OS median (months) or *X-years OS (%)*	ORR (%)	CR (%)	NCT (or eq.), [Ref]
Ibritumomab tiuxetan	Y-90	FL	II	74	1st line	–	–	+	+	40.2 *3y: 58 %*	NR *3y: 95 %*	94	58	NCT01493479, [40]
		FL	II	50	1st line	–	–	+	–	NR	NR	94	86	– [41]
		FL	II	59	1st line	–	–	+	–	25.9	NR	87	56	NCT00772655, [12]
		FL	I/II	47	Conditioning (R/R)	+ (C)	–	+	–	NR *3y: 85%*	NR *3y: 88%*	–	–	NCT00048737, [26]
		FL	II	61	Consolidation	+ (C)	–	+	–	NR *6y: 68%*	NR *6y: 93%*	–	38	– [10]
		FL	II	55	Consolidation	+ (C)	–	+	–	NR	NR	96	69	NCT00859001, [11]
		FL	II	52	Consolidation (R/R)	+ (C)	–	+	–	23.1	NR	98	30	NCT00637832, [46]
		FL	III	207	Consolidation	+ (C)	–	+	–	49.2	NR	81	59	NCT00185393, [45]
		FL	II	24	Consolidation (R/R)	+ (C)	–	+	–	NR *2 y: 59 %*	NR *2y: 95%*	88	83	– [47]
		FL	I	9	R/R	+ (C)	–	+	–	–	–	89	33	– [48]
		FL MZL	II	31	1st line	–	–	+	–	NR	NR	100	97	NCT00493467, [42]
		MZL	II	16	1st line	–	–	+	–	47.6 *5 y: 40%*	NR *5 y: 72%*	88	56	NCT00453102, [43]
		FL MZL	II	20	Consolidation	+ (C)	–	+	–	47.2	NR	100	79	– [13]
		MZL	P	30	R/R	–	–	+	–	NR	NR	90	77	– [50]
		MCL	Retro	46	Conditioning	+ (C)	Auto	+	–	45 *4 y: 41%*	NR *5 y: 71%*	100	100	– [27]
		MCL	II	56	Consolidation	+ (C)	–	+	–	–	94.8	82	55	– [15]
		MCL	II	46	Consolidation (1st line or R/R)	+ (C)	–	+/–	–	1st line: 39.6 R/R: 26.4	1st line: 78 R/R: 78	–	1st line: 91 R/R: 75	– [21]
		MCL	II	56	Consolidation	+ (C)	–	+	–	34.2	NR *5 y: 73%*	82	55	– [17]
		MCL	P	90	Consolidation (1st line: 50%, R/R: 50%)	+ (C)	–	+	–	25.3	48.6	89 51	1st line: 67 R/R:38	– [51]
		MCL	II	162	Late intensification	+ (C)	Auto	+	–	NR *4y: 71%*	NR *4y: 78%*	–	91	NCT00514475, [38]
		MCL Low-grade B-NHL	I	12	R/R	+ (C)	–	+	–	6.4	NR	50	42	– [52]
		DLBCL	Retro	63	Conditioning	+ (C + R)	Auto	–	–	NR *2 y: 68%*	NR *2 y: 90%*	–	–	– [39]
		DLBCL	II	30	Conditioning (R/R)	+ (C)	Auto	+	–	NR *3 y: 63%*	NR *3 y: 61%*	70	60	EudraCT 2007-003198-22, [28]
		DLBCL	II	11	Consolidation	+ (C)	Auto	+	–	– *2 y: 18%*	– *2y: 36%*	–	–	– [16]
		DLBCL	II	62	Consolidation (R/R)	+ (C)	–	+	–	FFS median: 15.1	23	50	31	– [22]
		Burkitt Lymphoma	R	27	Consolidation	–	–	+	–	NR *4 y: 95%*	NR *4 y: 95%*	–	–	– [53]
		B-NHL	II	20	Conditioning (R/R)	+(C)	Allo	+	–	*3 y EFS: 20%*	*3 y: 20%*	–	–	NCT00302757, [29]

		B-NHL	II	20	Conditioning (R/R)	+ (C)	Allo	+	−	12<br>*4 y: 44%*	−<br>*4 y: 44%*	71	64	NCT00644371, [30]
		B-NHL	II	122	Conditioning	+ (C)	Auto	+	−	NR	NR	−	36	NCT00695409, [33]
		B-NHL	II	40	Conditioning	+ (C + TBI)	Allo	+	+	−<br>*2.5 y: 31%*	−<br>*2.5 y: 54%*	−	7	NCT00119392, [34]
		B-NHL	Retro	18	Conditioning	+ (C)	Allo	+	−	*3 y: 71%*	*3 y: 87%*	−	−	− [35]
		B-NHL	CS	7	Consolidation (post ASCT)	−	Auto	+	−	−	−	−	71	− [54]
		B-NHL	I	30	R/R	+ (I)	−	+	+	42.7	NR	93	63	NCT00438880, [55]
		B-NHL	II	5	R/R	+ (C)	−	+	−	−	−	0	0	NCT01686165, [56]
		B-NHL	I	17	R/R	−	−	+	−	12.3	NR	82	35	NCT00033423, [57]
		MM	I	30	Conditioning	+ (C)	Auto	+	+	16.5	63.4	73	23	NCT00477815, [36]
Rituximab	I-131	FL	II	68	1st line	−	−	+	−	−	NR	99	82	Australian Clinical Trial Registry No. 12607000153415, [44]
		Low-grade (Ind.) and aggressive (agg.) B-NHL	Retro	36	R/R	−	−	+	−	−	−	Ind.:52.4<br>Agg.: 13.4	Ind.:14.3<br>Agg.: 6.7	− [59]
		B-NHL	I/II	23	Conditioning (R/R)	+ (C)	Auto	+	−	47.5	101.5	87	64	− [31]
		B-NHL	II	31	R/R	−	−	+	+	9.8	48.2	68	−	− [58]
	Lu-177	FL<br>MCL<br>Low-grade B-NHL	I/II	31	R/R	−	−	+	−	−	−	52	21	− [60]
		B-NHL	P	10	R/R	−	−	+	−	−	−	−	−	− [61]
	Y-90	B-NHL	CS	20	R/R	−	−	+	−	−	−	45	10	− [62]
Tositumomab	I-131	FL	III	532	Consolidation (1st line)	+ (C)	−	−	−	NR<br>*2 y: 80%*	NR<br>*2 y: 93%*	84	45	NCT00006721, [18]
		FL	III	531	Consolidation (1st line)	+ (C)	−	−	−	NR<br>*10 y: 49%*	NR<br>*10 y: 78%*	98	41	NCT00006721, [20]
		FL	III	14	R/R	−	−	+	−	NR	NR	−	43	NCT00268983, [49]
		Low-grade B-NHL	II	93	R/R	−	−	+	−	12	59.8	43	−	− [63]
		DLBCL	III	224	Conditioning (R/R)	+ (C)	Auto	−	−	−<br>*2 y: 47.9%*	NR<br>*2 y: 61%*	−	−	NCT00329030, [32]
		DLBCL	II	84	Consolidation (1st line)	+ (C)	−	+	−	NR<br>*2 y: 69%*	NR<br>*2 y: 77%*	86	61	NCT00107380, [19]
		DLBCL<br>MCL	II	39	Consolidation (1st line)	+ (C)	−	+	−	All: 66.5<br>DLBCL: 36.1<br>MCL: 69.4	All: 96.2<br>DLBCL: 36.1<br>MCL: *5 y: 79%*	92.3	82	− [23]
		B-NHL	I	36	Conditioning (R/R)	+ (C)	Auto	+	−	NR<br>3 y: 53%	NR<br>3 y: 54%	−	79	NCT00110071, [64]
		B-NHL	II	107	Conditioning (R/R)	+ (C)	Auto	+	−	*10y:*<br>*All: 61%*<br>*Low-grade: 64%*<br>*MCL: 43%*	*10y:*<br>*All: 62%*<br>*Low-grade: 71%*<br>*MCL: 48%*	−	82	− [37]
		B-NHL	CS	39	R/R	−	−	+	−	6.6	−	62	46	− [65]
		B-NHL	I	25	R/R	+ (C)	−	−	−	7	−	64	44	NCT00777114, [66]
		HL	I	12	R/R	−	−	−	−	−	−	−	−	− [67]

[1]B-NHL: B-cell non-Hodgkin lymphoma; DLBCL: Diffuse large B-cell lymphoma; FL: Follicular lymphoma; HL: Hodgkin lymphoma; MCL: Mantle cell lymphoma; MM: Multiple myeloma; MZL: Marginal zone lymphoma. [2]CS: Case series; P: Prospective; Retro: Retrospective. [3]*n*: Number of patients. [4]R/R: Relapsed/refractory; SCT: Stem-cell transplantation. [5](C): Chemotherapy; (I): Immunotherapy; (R): Radiotherapy; (TBI): Total-body irradiation. CR: Complete response; DFS: Disease-free survival; EFS: Event-free survival; FFS: Failure-free survival; NR: Non-reached; ORR: Overall response rate; OS: Overall survival. *Fractionation concerns RIT protoco.
